# Challenges of Tracheostomy in Patients Managed for Severe Tetanus in a Developing Country

**Published:** 2010

**Authors:** Ayotunde James Fasunla

**Affiliations:** 1 Department of Otorhinolaryngology, University College Hospital, Ibadan, Nigeria

**Keywords:** Complications, Developing country, Intubation, Tetanus, Tracheostomy

## Abstract

**Objectives:**

Severe tetanus is one of the indications for admissions into the intensive care unit requiring muscular paralysis, tracheostomy and mechanical ventilatory support. This study aimed to evaluate tetanus patients managed with tracheostomy and to determine associated complications.

**Methods:**

This was a 16-year review of patients who were managed for tetanus with tracheostomy in an intensive care unit between 1999 and 2009. The data collected from the medical records including demographic data, clinical presentations, tetanus immunization history, suspected portal entry of infection, duration of ICU admission, endotracheal intubation, tracheostomy, and complications.

**Results:**

Of the 31 patients studied, 11 (35.48%) were aged ≤15 years and all had history of completed childhood tetanus vaccination. Only 6 (19.35%) patients who were above 15 year-old had no history of previous childhood tetanus vaccination. None of these patients had tetanus booster shot. All patients had tracheostomy and 42% developed complications following tracheostomy.

**Conclusions:**

Tetanus is still a major health problem in developing countries and this can be prevented if recommended childhood tetanus vaccination and booster shots regimen are properly taken. Although, tracheostomy is associated with complications in severe tetanus patients, these patients would have all died of cardio-respiratory failure if tracheostomy had not been performed.

## INTRODUCTION

Tetanus is a vaccine preventable disease and it accounts for one of the indications for admission into the intensive care unit of hospitals.^1^,^2^ Despite the intention of World Health Organization to eradicate it by 1995, it still remains an important cause of death in most developing countries.^3^ It affects both the unvaccinated and inadequately vaccinated individuals.^2^,^4^ *Clostridium tetani*, an anaerobic, spore-forming, gram positive rod, is the infectious organism and its portal entry is usually an open skin wound which may be cephalic or non-cephalic in location.^5^ The exotoxins of these organisms are responsible for the clinical manifestations which include trismus, dysphagia, and muscular rigidity.^6^ Spasm related respiratory compromise, hospital acquired pneumonia and autonomic instability are usually the main causes of morbidity and mortality of this disease.^1^,^7^ Mechanical ventilation via intubation amidst other intensive care therapies (use of sedatives and nondepolarising neuromuscular blocking agents) may prevent sudden death from spasm related respiratory failure and significantly reduce mortality rate from this disease.^8^ Endotracheal intubation, as a method of an artificial airway, is usually preferred if the need will not exceed 10 days, otherwise, a tracheostomy tube is used especially when the need for an artificial airway is envisaged to exceed 21 days.^9^ As promising as these artificial airways are in preventing death due to asphyxia resulting from laryngeal muscle spasm (and acute airway obstruction), respiratory muscle spasm, aspiration or extreme fatigue, they may not be without challenges. This study therefore was carried out to evaluate patients with moderate and severe tetanus managed also with tracheostomy and to find out the problems associated with the tracheostomy.

## METHODS

This was a 16-year review of patients who had moderate or severe tetanus and were managed in intensive care unit of University College Hospital, Ibadan, Nigeria with tracheostomy between 1999 and 2009. These patients belonged to the stage 11 or 111 classification of tetanus according to the clinical staging system.^10^ The data collected from the medical records included demographic data, clinical presentations, tetanus immunization history, suspected portal entry of infection, duration of intensive care unit admission, duration of endotracheal intubation before tracheostomy, duration of tracheostomy and complications. The results were prepared in tables and simple descriptive forms. The statistical analysis was performed using statistical package for social sciences version 11.

## RESULTS

Thirty one patients diagnosed and managed for moderate or severe tetanus with tracheostomy were reviewed. There were 21 (67.74%) males and 10 (32.26%) females with a ratio of 2:1. Their age ranged from 8 to 66 years with a mean age of 31.04 years. Eleven patients were aged ≤15 years and all had history of completed childhood tetanus vaccination. However, of the remaining 20 (64.52%) patients who were above 15 years of age, only 6 (19.35%) patients had no history of previous childhood tetanus vaccination. None of the patients in this study reported a history of previous tetanus vaccination within the last 5 years of presentation. The portal entry was cephalic in 8 (25.81%) patients and non-cephalic in 19 (61.29%) patients. However, 4 (12.90%) patients had either no obvious wound or the portal entry could not be determined. All the cephalic portal entries were otogenic and 75% of them were 15 years of age and below. The remaining 19 (61.29%) patients that their portal entries could be identified had wound which ranged from minor skin abrasions to foot puncture by nail or lacerations. The incubation period (time from inoculation of *Clostridium tetani* to the first symptom) could only be ascertained in these patients and it ranged from 3 to 18 days with the mean of 8.4 ± 4.2 days. In 13 (68.42%) patients, the incubation period was less than 8 days. The period of onset of tetanus (time from the first symptom to the first generalized spasm) ranged from 3 to 8 days with the mean of 4.6 ± 3.2 days. All the patients presented with classical symptoms of trismus, rigidity and generalized spasms. All these patients were managed with tetanus toxoid, human tetanus immunoglobulin, antibiotic therapy, wound care, muscle relaxants, sedatives and artificial ventilation. They all had prior endotracheal intubation for a period of 5 to 18 days before tracheostomy. Cuffed portex tracheostomy tube was used initially for all patients. This was thereafter changed to plain portex tracheostomy tube (with inner tube) when their spasms have been significantly controlled. This period ranged from 6 to 14 days. The duration of intensive care unit admission ranged from 20 to 31 days with the mean of 24 ± 4.6 days. The duration of tracheostomy ranged from 3 weeks to 4 years. The complications observed are as shown in Table 1. Nine (29.03%) patients died while still being managed in the intensive care unit, 8 to 13 days post-admission.

**Table 1:** Complications of tracheostomy in patients with severe tetanus.

Complications	Frequency	Percentage (%)
Suprastomal granulation tissues	5	38.46
Pneumonia	4	30.76
Tracheal stenosis	1	7.69
Persistent tracheocutaneous fistula	1	7.69
Severe stomal infection	1	7.69
Impacted distal end of tracheostomy tube	1	7.69
Total	13	100.00

## DISCUSSION

**Figure 1:**
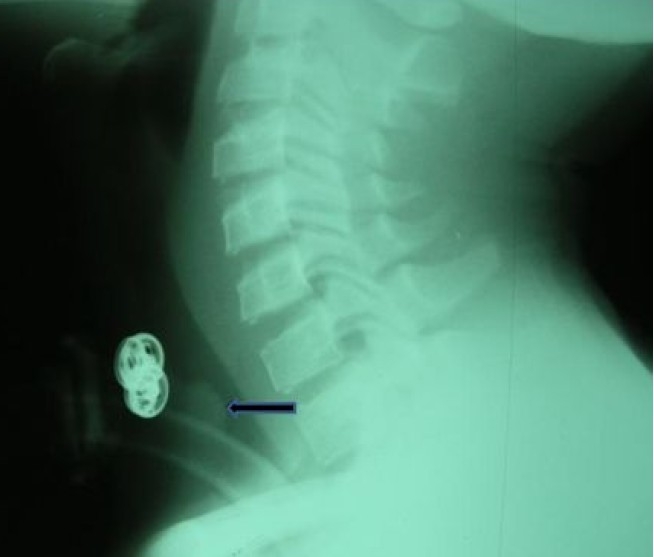
Lateral soft tissue in neck x-ray showing the shadow of tracheostomy tube in-situ and suprastomal soft tissue shadow [granulation tissue (arrow)] with narrowing of the tracheal air column at the level in a patient managed for severe tetanus.

Tetanus still constitutes a major health challenge and it is an important cause of preventable death in developing countries.^3^ Because there is essentially no natural immunity to tetanus toxin, the only effective way to prevent tetanus is by prophylactic immunization. It is therefore very important, in order to have protection against tetanus, that all age groups have the universal primary immunization with subsequent maintenance of adequate antitoxin levels by means of appropriately timed boosters.^11^ This will, in no doubt, prevent people from developing tetanus as well as morbidity and mortality which usually follow it. In some countries with good primary immunization programs, people may still be vulnerable, either because of incomplete primary vaccination, use of poorly preserved vaccines (defective cold chain system) or because protective antibody levels against tetanus in these patients had declined over time.^12^,^13^ It thus means that tetanus could in theory be eradicated from the world, but realistically this is not going to happen even with an already available successful implementation of the prevention programs especially in developing countries. In the developed or industrialized world, the incidence of tetanus has drastically reduced with only few elderly individuals being affected by the disease because they lack or have insufficient immunity against tetanus.^14^ However, tetanus is still a major health problem in developing or underdeveloped countries^15^ and this study showed that all age groups are still being affected by the disease. Although more than 80% of these patients had history of completed recommended tetanus vaccination during childhood, they still developed tetanus. The reason for this may be that these patients did not actually complete the vaccinations regimen as there were actually no records of immunization to verify this at presentation. It could also be that the tetanus vaccines which they received have become ineffective and impotent due to defective cold chain system. This might be the situation, especially in the country where this study was carried out, because of lack of constant supply of electricity leading to defective storage condition. When these vaccines are not properly stored under an ideal temperature, they become denatured and lose their potency and ability to confer the expected immunity. Open wound contamination or infection by *Clostridium tetani* is usually the cause of tetanus. In unvaccinated individuals or people with waning immunity, minor wounds could become contaminated with *Clostridium tetani* and cause this fatal disease.^11^ The tetanus toxoid vaccine may exist alone however; tetanus vaccine is given usually to children as part of the diphtheria and tetanus toxoids and pertussis (DTP) shot.^16^ Sometimes, the childhood combination vaccine formulation may include diphtheria and tetanus toxoids and acellular pertussis with Haemophilus influenza type b vaccine (HTaP-Hib) as well as polyribosylribitol phosphate polysaccharide conjugated to tetanus toxoid and H. influenza type B (PRP-T Hib).^11^ The advantages of the combination vaccine include overcoming the challenges of multiple injections, especially in children who are behind schedule in vaccination as well as improve the timely vaccination coverage. The complete dose is usually completed before the age of 6 years. However, this has been proven not to give a lifelong protection against tetanus. It is therefore recommended that adolescents should get a booster shot between the ages of 11 and 18 years, and that adults should receive a tetanus booster shot every 10 years. 16 Unfortunately, none of the patients in this study ever received the tetanus booster shot at any time. This might also explain their vulnerability to the disease. The suspected portal entry for *Clostridium tetani* was identified in 87% of these patients and in 25.81% it was cephalic route. The cephalic part of the body normally does not readily come in contact with the soil where *Clostridium tetani* inhabit. However, inoculation is possible especially in situation where objects such as fingers, broom sticks, pen covers, etc., which had been contaminated with *Clostridium tetani*, are inserted into the ear. Self-medication with local concoctions, contaminated by the feces of man or domestic animals where Clostridium tetani also inhabit, dropped into the ear with the intention of treating a discharging ear may also be the source of inoculation of *Clostridium tetani* in the patients with otogenic tetanus. This habit is common in the rural areas of the country of this study where majority of these patients reside. This should therefore be discouraged through health education and the risk of developing tetanus amidst other problems emphasized. Open wounds should be kept clean and properly managed. The short incubation period in 70% of these patients, shorter onset time of tetanus and closeness of the portal entry to the brain in 25% are identifiable factors associated with a more severe disease in this study. The clinical features of tetanus seen in these patients did not differ from what had been reported before.^1^,^3^,^4^,^6^ These presentations usually arise from the action of tetanus toxin, which blocks inhibitory input of gamma aminobutyric acid to motor neurons, resulting in unchecked motor nerve activity.^17^ Muscle tone is increased, producing the characteristic trismus, “risus sardonicus” and opisthotonus. Generalized spasms typically develop one to four days after the initial symptoms and if frequent or prolonged, might impede respiration. In severe tetanus, the autonomic nervous system is affected, which results in marked cardiovascular instability with rapidly fluctuating blood pressure. These patients will require artificial respiratory supports for the period of active symptoms to prevent cardio-respiratory failure and death.^15^,^18^ Tracheostomy was performed in all patients in this study and they all had artificial respiratory support. Twenty nine percent (five adults and four children) of these patients did not survive the illness. The cause of death in one of the adult patients was adult respiratory distress syndrome. The remaining adult patients and a child died of sympathetic instability while the other children died from severe sepsis. The deaths in this study were not directly attributable by tetanus or tracheostomy because they may also arise in any other severely ill patients who are paralyzed and on mechanical ventilator support. However, the potential source of sepsis in these patients could still be the tracheostomy wound. Tetanus patients with the severe form of the disease were managed in our institution with deep sedation, muscular paralysis and mechanical ventilation in the intensive care unit. Endotracheal intubation was the preferred initial artificial airway in all these patients. However, this was converted to tracheostomy when the episodes of spasms did not decline at about the 10^th^ day or when there was frequent endotracheal tube blockage or when the patient clinical conditions became worse. All the patients in this study had tracheostomy at different periods following endotracheal intubation. The use of tracheostomy in the management of patients with severe tetanus will undoubtedly prevent death due to asphyxia from laryngeal muscle spasm (and acute airway obstruction), respiratory muscle spasm and aspiration. However, this tracheostomy may be associated with complications and some of which are well-relevant even after the placement of the tracheostomy tube. Complications of tracheostomy for various indications have been documented in the literature.^19^–^22^ The complications of tracheostomy in tetanus patients in this series are presented in Table 1. However, complications due to surgical operation, displacement of the tube and tube blockage by mucoid secretions and crusts are excluded from the list. This is because these are common and frequent with most patients on tracheostomy. They were easily identified and corrected. About 42% of these patients developed one form of complication or the other. Suprastomal granulation tissue (Figure 1) accounted for 38% of complications of tracheostomy in this series. All these patients had prior intubation with polyvinyl chloride cuffed orotracheal tube for 5 to 18 days in the intensive care unit of our hospital before tracheostomy.

**Figure 2:**
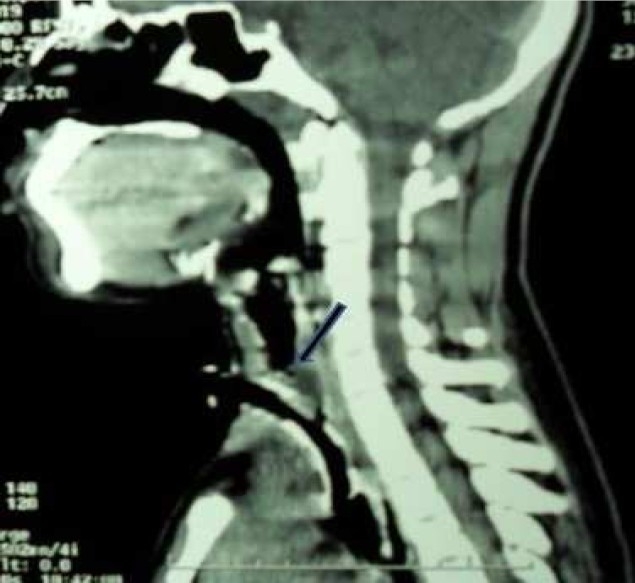
A sagittal reconstruction of computerized tomographic scan of the neck with tracheostomy tube in situ and tracheal stenosis (arrowed) in a patient managed for severe tetanus.

There was a significant correlation between the duration of prior orotracheal intubation and suprastomal granulation tissue formation (P=0.000). Rough intubation, use of an inappropriate large size endotracheal tube and chewing on the orotracheal tube during uncontrolled spasm may cause laryngotracheal mucosal tear.^22^–^24^ This may heal with polyp or exuberant granulation tissue which then projects into the laryngotracheal lumen and narrows the airway. This is usually unnoticed until during decannulation process which becomes difficult. When pretracheostomy intubation is desirable in tetanus patients, careful nasotracheal intubation with appropriate size tube and adequate muscle relaxation are recommended. A poor surgical technique during tracheostomy that injures and exposes the mucosa overlying the tracheal cartilage and continuous irritation of the tracheostomy wound amidst other factors such as infection, repeated trauma, etc., may promote suprastomal granulation tissue formation. One patient developed tracheal stenosis at the site of tracheostomy tube cuff. The stasis of secretion around and above the cuff encourages bacterial infection. These bacteria and their released toxins may inflict damage on the tracheal mucosa and underlying cartilage causing chondritis. This usually promotes formation of granulation tissue during healing process. In addition, the mechanical irritation on the tracheal mucosa by the tracheostomy cuff during uncontrolled spasm in a patient with severe tetanus may promote exuberant granulation tissue formation at this site and eventually tracheal stenosis.^22^,^23^ This is the situation in the index patient with tracheal stenosis from this series. The presentation was noticed during decannulation process which was difficult. A flexible tracheoscopy was performed and a ring granulation tissue was identified at the area of the tracheostomy cuff. A microlaryngoscopy and tracheoscopy was later performed to endoscopically remove the circumscribed obstructive granulation tissue in the trachea by electrocautery and then, surgical decannulation was successfully performed. Other possible reasons while tracheal stenosis (Figure 2) could develop in tetanus patients include if the cricoid cartilage is injured during the surgery of tracheostomy and the mucosa overlying the cricoid cartilage is exposed.^24^,^25^ In addition, the use of inappropriately large size tracheostomy tube will put pressure on the anterior wall of the trachea thereby causing its ischaemic injury and weakness (tracheomalacia) with significant narrowing of tracheal lumen at the site. This also may result in failure of decannulation process. Four patients developed pneumonia in this series.

A tracheostomized patient who is paralyzed has lost the natural ability to clear the tracheobronchial tree of secretion. This may lead to stasis and retained secretions in the chest. The secretions may become infected and result in pneumonia. The continual struggle against retained secretions in these patients could be overcome if the breathing air is humidified and the tracheobronchial tree is suctioned regularly as required with an appropriate size, sterile soft rubber catheter through the tracheostomy tube. Chest physiotherapy and sensitive antibiotic may be required to treat this complication. Other complications seen in this series included severe tracheostomy infection, persistent tracheocutaneous fistula and impacted dislodged tip of tracheostomy tube. The severe stomal infection was marked by painful swelling at the tracheostomal site with purulent greenish discharge from and around the tracheostomy tube. The culture result from the tracheal swab from this patient showed *Pseudomonas aeruginosa*. This was thought to be a nosocomial infection and it responded well to antibiotic therapy. This organism has been reported to cause tracheostomy wound and bronchopulmonary infection in the literature.^26^ The patient with persistent tracheacutaneous fistula had secondary closure of the wound after complete excision of the fistula. The impacted distal tip of the tracheostomy tube in a patient from this study is an uncommon and unusual complication of tracheostomy tube. The exact reason for the breakage of the tracheostomy tube and dislodgment of the distal end into the distal tracheal lumen could not be ascertained. However, this may be because the patient had worn the metal tracheostomy tube for a period of 7 months without a change to a new tube for financial reason. The tube might have become weak and corroded. It was however removed with forceps during bronchoscopy.

## CONCLUSION

In conclusion, tetanus is still a major health problem in developing countries and the incidence can only be reduced if complete recommended childhood tetanus vaccinations and booster shot regimens are appropriately and adequately taken. Health education on personal hygiene and importance of tetanus vaccinations and the risk involved, if not taken should be emphasized. Although, tracheostomy is associated with complications in severe tetanus patients, these patients would have died of asphyxia and cardiorespiratory failure if tracheostomy had not been performed. It is therefore justified to perform tracheostomy on patients with moderate to severe tetanus in order to prevent sudden death which may occur from laryngeal and respiratory muscle spasms.
